# CCAT2 enhances autophagy‐related invasion and metastasis *via* regulating miR‐4496 and ELAVL1 in hepatocellular carcinoma

**DOI:** 10.1111/jcmm.16859

**Published:** 2021-08-19

**Authors:** Jing Shi, Cao Guo, Junli Ma

**Affiliations:** ^1^ Affiliated Hospital of Jining Medical University Jining China; ^2^ Institute of Medical Sciences Xiangya Hospital Central South University Changsha China

**Keywords:** autophagy, CCAT2, hepatocellular carcinoma, metastasis

## Abstract

Autophagy is thought to contribute to the pathogenesis of many diseases, including cancer. Long non‐coding RNA (lncRNA) CCAT2 functions as an oncogene in a variety of tumours. However, it is still unknown whether CCAT2 is involved in autophagy and metastasis of hepatocellular carcinoma (HCC). In our study, we found that lncRNA CCAT2 expression was significantly increased in HCC tissue and was correlated with advanced stage and venous invasion. Further experiments revealed that CCAT2 induced autophagy and promoted migration and invasion *in vitro* and *in vivo*. Mechanistic investigations found that CCAT2 involved in HCC by regulating miR‐4496/Atg5 in cytoplasm. In nucleus, CCAT2 bound with ELAVL1/HuR to facilitate HCC progression. Our findings suggest that CCAT2 is an oncogenic factor in the progression of HCC with different regulatory mechanisms and may serve as a target for HCC therapy.

## 1 INTRODUCTION

Hepatocellular carcinoma (HCC) is one of the most common causes of cancer‐related death worldwide with rising incidence and mortality.^1^ Although therapeutic strategies including surgery and interventional therapy have improved the survival of HCC patients, HCC is still a highly fatal tumour, with aggressive malignancy and late diagnosis. Therefore, investigations and identification of the molecular mechanisms underlying progression of HCC can help to develop the novel prognostic biomarkers and therapeutic targets and thus are clinically important.

Autophagy is a catabolic reaction to stress in cells.^2^ Unbalance of autophagy is closely related to many diseases, including neurodegeneration, muscular disorder and cancer.^3^ The role of autophagy in cancer‐related processes is under investigation. Studies showed that autophagy can promote or suppress cancer progression during different periods of tumour progression.^4^, ^5^ The characteristics of autophagy have been extensively investigated; however, the specific roles and mechanism of autophagy in HCC are still not well known. Therefore, a comprehensive understanding of autophagy pathways involved in HCC is of important meaning.

Long non‐coding RNAs (lncRNAs) are classified as a sort of RNA with transcripts longer than 200 nucleotides. Although lncRNAs hold no or limited protein‐coding ability, they can take part in various biological processes of many diseases, including HCC.^6^, ^7^ Dysregulation of lncRNAs has been frequently reported to play important roles in the development of cancerous alterations, including cellular tumorigenesis, apoptosis, invasion or autophagy.^8^ Non‐coding RNAs can act as regulators of autophagy process.^9^ Recently, HCC‐related lncRNAs with oncogenic or tumour‐suppressive roles that are involved in the pathogenesis of HCC are also defined.^7^, ^10^

Colon cancer‐associated transcript 2 (CCAT2), located on chromosome 8q24.21, originally identified as an oncologic lncRNA in colorectal cancer.^11^ Subsequent studies have reported that CCAT2 was abnormally expressed in multiple types of cancer including breast cancer,^12^ myeloid malignancies^13^ and HCC^14^ and related to tumorigenesis and progression. However, the exact functions of CCAT2 in HCC remain unclear. To identify the roles of CCAT2 as a potential tumour marker and provide the basis for prognosis prediction in HCC, we explored the functions and involved molecular mechanisms of CCAT2 in HCC metastasis *in vitro* and *in vivo*.

ELAVL1/HuR, one of the ELAV/Hu family, is a RNA‐binding posttranscriptional regulator.^15^ ELAVL1 stabilizes target mRNAs or promotes their translation and nucleocytoplasmic translocation.^16^ By targeting mRNAs, ELAVL1 involves in cell proliferation, apoptosis and differentiation.^17^ ELAVL1 is a cancer‐related RNA‐binding proteins (RBPs) and plays an oncogenic role in the progression of various cancers.^18^, ^19^ For HCC, the roles of ELAVL1 have also been investigated. ELAVL1 facilitates progression of HCC by stabilizing some oncogenes, such as β‐catenin and ETS1.^20^, ^21^, ^22^ In our study, we found that CCAT2 was significantly upregulated in HCC tissues and could regulate cell migration both *in vitro* and *in vivo*. Further investigations found that CCAT2 could exhibit different regulatory mechanisms by miR‐4496 and ELAVL1 regulation in HCC.

## 2 MATERIALS AND METHODS

### 2.1 HCC patients and tissue samples

A total of 61 pairs of tumour and adjacent non‐tumour were randomly collected from HCC patients who received liver resection from May 2011 to December 2012. The samples were snap‐frozen in liquid nitrogen and transferred to the −80°C refrigerator for subsequent experiments. The collected samples were confirmed by two experienced pathologist. The clinical data of HCC patients including tumour‐node metastasis (TNM) staging were also collected.

### 2.2 Cell culture and transfection

Human HCC cell lines of HepG2 and HCCLM3 were purchased from Procell (Wuhan, China) and cultured in high glucose Dulbecco's modified Eagle's media supplemented with 10% foetal bovine serum (FBS) (GIBCO, Grand Island, NY) at 37°C with 5% CO_2_. HepG2 and HCCLM3 cells were stably transfected with lentivirus‐based short hairpin RNA (shRNA) targeting CCAT2 according to the manufacturer's instructions. The sequences of shRNA are listed in Table S1. Regulated expression of CCAT2 was confirmed by quantitative real‐time PCR (qRT‐PCR).

### 2.3 Transwell invasion assays

Hepatocellular carcinoma cells were re‐suspended in serum‐free medium and seeded in the upper chambers, while the lower chambers were covered with DMEM containing 10% FBS. After incubation for 24 h, the invaded cells were fixed with 4% paraformaldehyde and stained with 0.1% crystal violet. The stained cells were counted under an inverted microscope to evaluate HCC cells' invasive ability.

### 2.4 Wound healing

Appropriate HCC cells were seeded into 6‐well plates and cultured. The confluent cell monolayers were scratched by a 200‐μl pipette tip, washed with PBS and cultured in serum‐free medium for 24 h. The speed of wound closure was imaged by ImageJ.

### 2.5 RNA isolation and qRT‐PCR

Total RNA was extracted from tumour tissue or cells by Trizol reagent (Invitrogen, Carlsbad, CA) according to the manufacturer's protocol. qRT‐PCR was performed in triplicate using SYBR Green fluorescent‐based assay (TaKaRa Bio Inc., Otsu, Japan). Relative mRNA expression levels were calculated based on the threshold cycle (Ct) values as: 2^−ΔCt^ [ΔCt = Ct (targeting gene)–Ct (GAPDH)] and were normalized. The relative RNA expression levels were calculated with the 2^−ΔΔCt^ method and normalized to the internal control of GAPDH. The primer sets are listed in Table S2.

### 2.6 Western blot

Total protein was separated by sodium dodecyl sulphate‐polyacrylamide gel electrophoresis (SDS‐PAGE) and transferred onto PVDF membranes (Millipore, Bedford, MA). Then, the blotted membranes were blocked in 5% skim milk and incubated with the primary antibodies at 4°C overnight. HRP‐conjugated IgG (KPL, Gaithersburg, MD) was used as the secondary antibody. GAPDH/β‐actin protein expression was determined as an internal control. The bands were visualized by the enhanced chemiluminescence kit on a ChemiDoc XRS+system (Tanon, Shanghai, China).

### 2.7 Transmission electron microscopy (TEM)

A transmission electron microscope was used to observe the autophagosomes of HCC cells. The treated cells were fixed using 2.5% glutaraldehyde and treated with 1% osmium tetroxide for 3 h. After being dehydrated in a graded series of ethanol baths, the samples were infiltrated and embedded in Epon resin. Ultrathin sections (70 nM) were cut, stained with uranyl acetate and examined by a transmission electron microscope (JEM‐1230, Tokyo, Japan).

### 2.8 Adenovirus transfection and autophagic flux analysis

To observe the autophagic flux, mRFP‐GFP‐LC3 adenovirus (HanBio Technology Co., Shanghai, China) were transfected into HCC cells according to the manufacturer's instructions. Approximately 60% confluence, the treated cells were incubated with mRFP‐GFP‐LC3 adenovirus in growth medium at 30 multiplicities of infection (MOI). After fixed and washed, the imaging of autophagic flux in the transfected cells was captured by an Opera High Content Screening System (Perkin‐Elmer).

### 2.9 Luciferase reporter assay

Two luciferase reporters containing wild or mutant type were constructed to validate the interaction between miR‐4496 and CCAT2. The sequence of 3′‐UTR of CCAT2 was amplified from human genomic DNA. The luciferase reporter systems and miR‐4496 mimics or negative control were cotransfected. Luciferase activity was detected 48 h later according to the dual‐luciferase reporter assay protocol (Promega, Madison, WI). The relative luciferase activity was calculated by the ratio of firefly luciferase activity to renilla luciferase activity.

### 2.10 RNA pull‐down assay

Briefly, cell extract (2 μg) was mixed with biotinylated RNA. The streptavidin agarose beads (100 ml) were added to each binding reaction and incubated at room temperature for 1 h. Beads were washed and boiled in SDS buffer, and the retrieved protein was determined by Western blot.

### 2.11 RNA immunoprecipitation (RIP) assays

Cells were lysed and incubated with RIP buffer containing magnetic beads conjugated to human anti‐ELAVL1 antibody or negative control IgG. Then, the immunoprecipitated RNA was isolated and subjected to qRT‐PCR analysis.

### 2.12 Subcutaneous HCC tumour model in nude mice

Briefly, 5 × 10^6^ HCCLM3 cells transfected with si‐CCAT2 or si‐NC were injected subcutaneously into the nude mice (4 weeks old, male) respectively. Tumour growth was monitored until 4 weeks, and the tumour volume was calculated as volume (mm^3^) = (length × width^2^) × 0.5. To generate liver and lung metastasis models, the cells (3 × 10^6^/ml, 0.10 ml per mice) were injected into the tail vein of the nude mice. After 3 weeks, lung and liver were harvested when the mice were killed and subjected to haematoxylin‐eosin staining to analyse intrahepatic and pulmonary metastases. The animal experiments were approved by the Animal Use Committee of Jining Medical University, and all mice were treated humanely during the whole study period.

### 2.13 Immunohistochemistry (IHC)

The paraffin‐embedded sections were de‐paraffinization, dehydration, antigen retrieval and incubated with the primary antibodies against ELAVL1 (1:400 dilution, Abcam, Cambridge, UK) at 4°C overnight, followed by the secondary antibody. The IHC staining was scored according to the staining intensity and percentage of positive‐staining cells.

### 2.14 Statistical analysis

Statistical analysis was analysed by SPSS, version 17.0 (SPSS, Inc, Chicago, IL). Student's *t* test was used to analyse differences between two groups, and two‐way ANOVA was applied when more than two groups were compared. *p* < 0.05 was considered to be statistically significant.

## 3 RESULTS

### 3.1 CCAT2, overexpressed in tumour tissue, was significantly correlated with advanced stage and venous invasion of HCC

**FIGURE 1:**
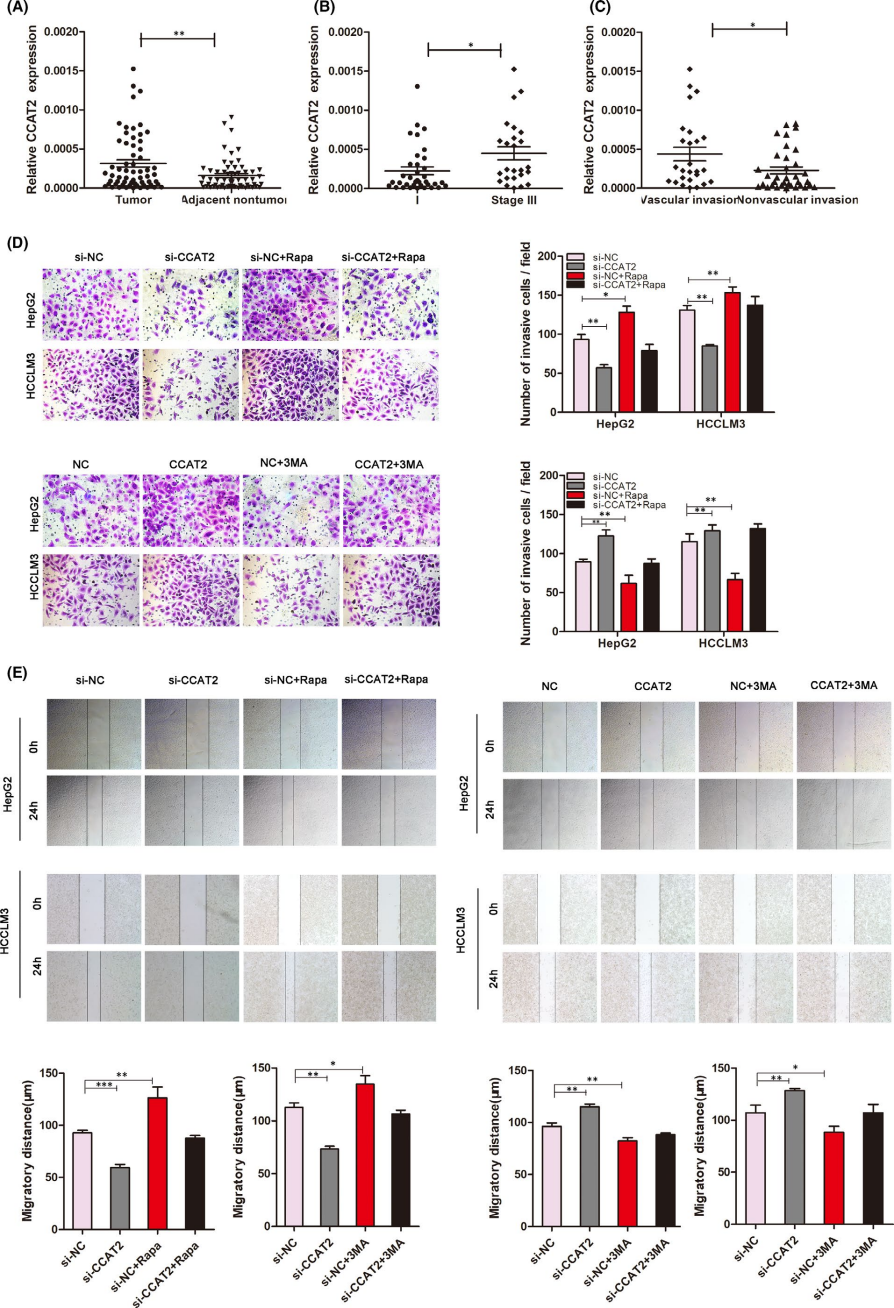
CCAT2 is associated with advanced stage and vascular invasion of HCC and promotes migration and invasion of HCC cells. (A) CCAT2 expression levels in tumour tissues were higher than the matched normal tissues (*p* < 0.001). (B) The patients with I or II stage exhibited higher CCAT2 expression in tumour tissues than those with stage III. (C) CCAT2 expression levels were higher in HCC with vascular invasion. (D&E) Transwell and wound healing assays indicated that CCAT2 promoted numbers of invasive cells and the rate of migration and 3‐MA inhibited these invasive and migratory abilities, while rapamycin could reincrease the invasive and migratory abilities of HepG2 and HCCLM3 cells inhibited by CCAT2 knockdown. ^**^ *p* < 0.01, ^***^ *p* < 0.001

We noticed that CCAT2 was overexpressed in HCC by using microarray (our unpublished data). We further evaluated the expression of CCAT2 in 61 pairs of frozen HCC and the adjacent tissues by qRT‐PCR. The results indicated that CCAT2 expression levels in HCC tumour tissues were significantly higher than the corresponding adjacent tissues (*p* < 0.005, Figure 1A). Furthermore, HCC patients at stage III exhibited higher CCAT2 expression levels in tumour than those at stages I/II (0.00045 ± 0.00008 *vs*. 0.00022 ± 0.00005, *p* < 0.05, Figure 1B). In addition, CCAT2 expression levels were statistically higher in HCC that displayed vascular invasion than those without invasion (0.00044 ± 0.00009 *vs*. 0.00023 ± 0.00004, *p* < 0.05, Figure 1C). It is suggested that increased CCAT2 expression was significantly correlated with TNM stage and the presence of venous invasion, while there was no significant association between CCAT2 and other clinicopathologic parameters such as age, gender, serum AFP, liver cirrhosis, tumour size and tumour number.

### 3.2 CCAT2 promotes migration and invasion of HCC cells *in vitro*

Our samples from patients with HCC indicated that CCAT2 might act as an oncogene. Furthermore, we used two HCC cell lines (HepG2 and HCCLM3) for *in vitro* research. HepG2 and HCCLM3 cells were, respectively, transfected with lentivirus‐based shRNA to knock down CCAT2 expression. As shown in Figure S1, si‐2 group exhibited more reduced CCAT2 expression and selected to be the targeted shRNA. Transwell invasion and wound healing assays were used to demonstrate that knockdown of CCAT2 reduced the migratory and invasive rate of HCC cells significantly and upregulation of CCAT2 showed the opposite trend (Figure 1D,E, *p* < 0.01). Furthermore, we found that these migratory and invasive capacities could also be influenced by 3‐MA, the autophagy inhibitor, with reduced rates. While rapamycin, the autophagy activator, could reincrease the migratory and invasive abilities of HepG2 and HCCLM3 cells inhibited by CCAT2 knockdown (Figure 1D,E), the results indicated that autophagy might involve in HCC progression.

### 3.3 CCAT2 induced autophagy in HCC cell lines

**FIGURE 2:**
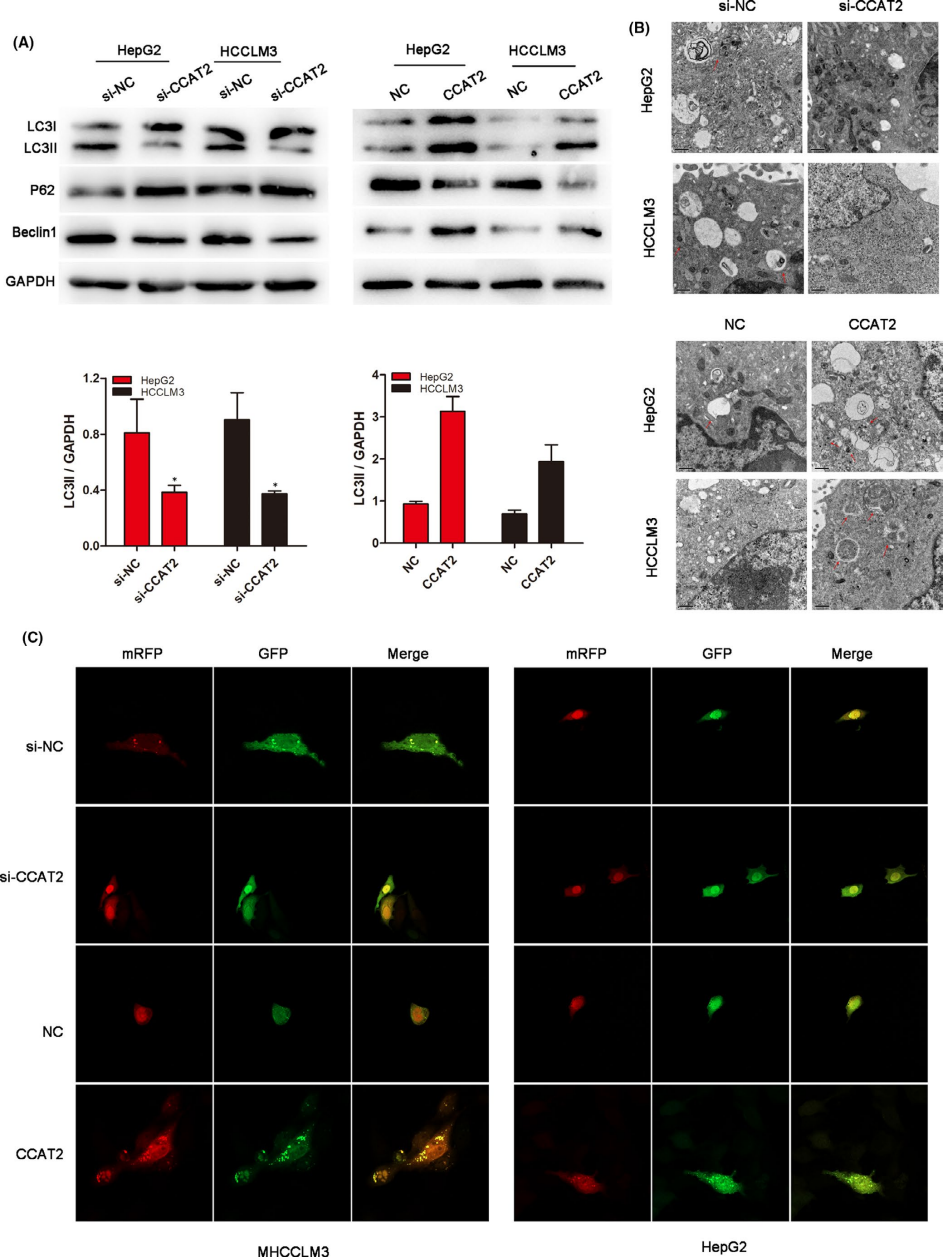
CCAT2 induced autophagy in HCC cell lines. (A) Western blot was used to detect the expression of P62, Beclin1 and conversion of LC3‐I to LC3‐II influenced by CCAT2. (B) Representative images of autophagosome/autolysosome (red sword) detected by TEM in HCC cells (original magnification: ×2000). Overexpressed CCAT2 promoted autophagy formation. (C) Accumulation of fluorescent cells with LC3 dots was detected by confocal microscope and CCAT2 induced more LC3 dots (*p* < 0.01, respectively). Data are shown as mean ± SD and from three independent experiments. ^*^ *p* < 0.05, ^**^ *p* < 0.01

To investigate the role of autophagy in HCC migration and invasion, we conducted the experiments to explore the effects of CCAT2 on autophagy. Autophagy‐related proteins were detected by Western blot assay when CCAT2 was regulated. As shown in Figure 2A, a decreased conversion of LC3‐I to LC3‐II was found in the si‐CCAT2 group, while when CCAT2 was upregulated, LC3‐II conversion was increased. The LC3‐II/GAPDH ratio, which was calculated to reflect autophagy activity, was also significantly decreased or increased according to CCAT2 expression (*p* < 0.05). Consistent with the change of LC3‐II, the expression of Beclin1 was also decreased after CCAT2 was reduced and increased when CCAT2 was upregulated, while the level of autophagic protein p62 exhibited the opposite trend. TEM was further performed to observe the autophagy activation and the result indicated decreased numbers of double‐membrane structures resembling autophagosomes when CCAT2 was knocked down and increased when CCAT2 was upregulated (Figure 2B). What's more, mRFP‐GFP‐LC3 puncta distributions were remarkably decreased in CCAT2‐reduced cells and increased in CCAT2‐upregulated cells, as compared with the negative control groups (Figure 2C). The results suggested that autophagy might involve in CCAT2‐promoted migration and invasion of HCC.

### 3.4 CCAT2 promotes metastasis of HCC cells *in vivo*

**FIGURE 3:**
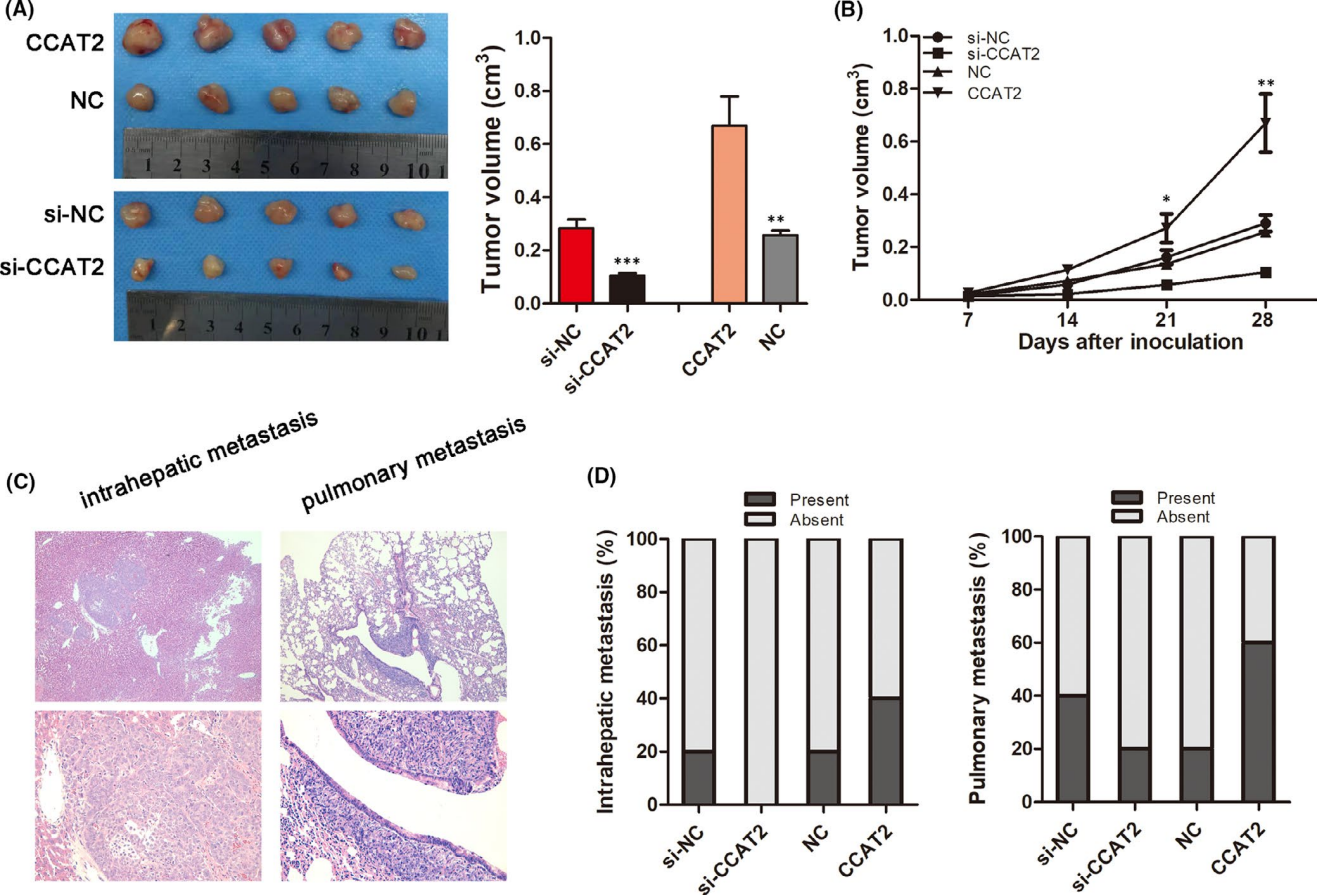
CCAT2 promoted metastasis of HCC cells *in vivo*. (A) Four weeks after subcutaneous implantation, the average tumour volume was smaller when CCAT2 was knocked down and larger when CCAT2 was upregulated (0.2839 ± 0.0321 cm^3^ vs. 0.1044 ± 0.0103 cm^3^, *p* < 0.001; 0.2568 ± 0.0178 cm^3^ vs. 0.6696 ± 0.1098 cm^3^, *p* < 0.01). (B) Tumour growth curves of subcutaneous implantation models of HCC. (C) Haematoxylin‐eosin staining of metastatic lung and liver of nude mice (original magnification: ×50 and 200). (D) The rate of intrahepatic metastasis (20% (1/5) *vs*. 0% (0/5); 20% (1/5) *vs* .40% (2/5) ) and pulmonary metastasis (40% (2/5) *vs*. 20.0% (1/5); 20% (1/5) *vs*. 60% (3/5)) were lower in CCAT2 knocked‐down group and higher in the overexpressed groups. ^*^ *p* < 0.05, ^***^ *p* < 0.001

To further explore the effects of CCAT2 on HCC *in vivo*, the stably knocked‐down or upregulated HCCLM3 cells were used to construct the subcutaneous and metastatic *in vivo* tumour models. We found decreased tumour growth rate and final smaller tumour size when CCAT2 was knocked down, while overexpressed CCAT2 promoted tumour growth (0.2839 ± 0.0321 cm^3^ vs. 0.1044 ± 0.0103 cm^3^, *p* < 0.001; 0.2568 ± 0.0178 cm^3^ vs. 0.6696 ± 0.1098 cm^3^, *p* < 0.01; Figure 3A,B). Moreover, the incidences of both intrahepatic (20% (1/5) *vs*. 0% (0/5)) and pulmonary metastasis (40% (2/5) *vs*. 20.0% (1/5)) were lower when CCAT2 was knocked down, and higher in the CCAT2 overexpressed groups (20% (1/5) *vs* .40% (2/5); 20% (1/5) *vs*. 60% (3/5); Figure 3C,D).

### 3.5 CCAT2 promotes HCC cell migration and invasion by miR‐4496 in cytoplasm

**FIGURE 4:**
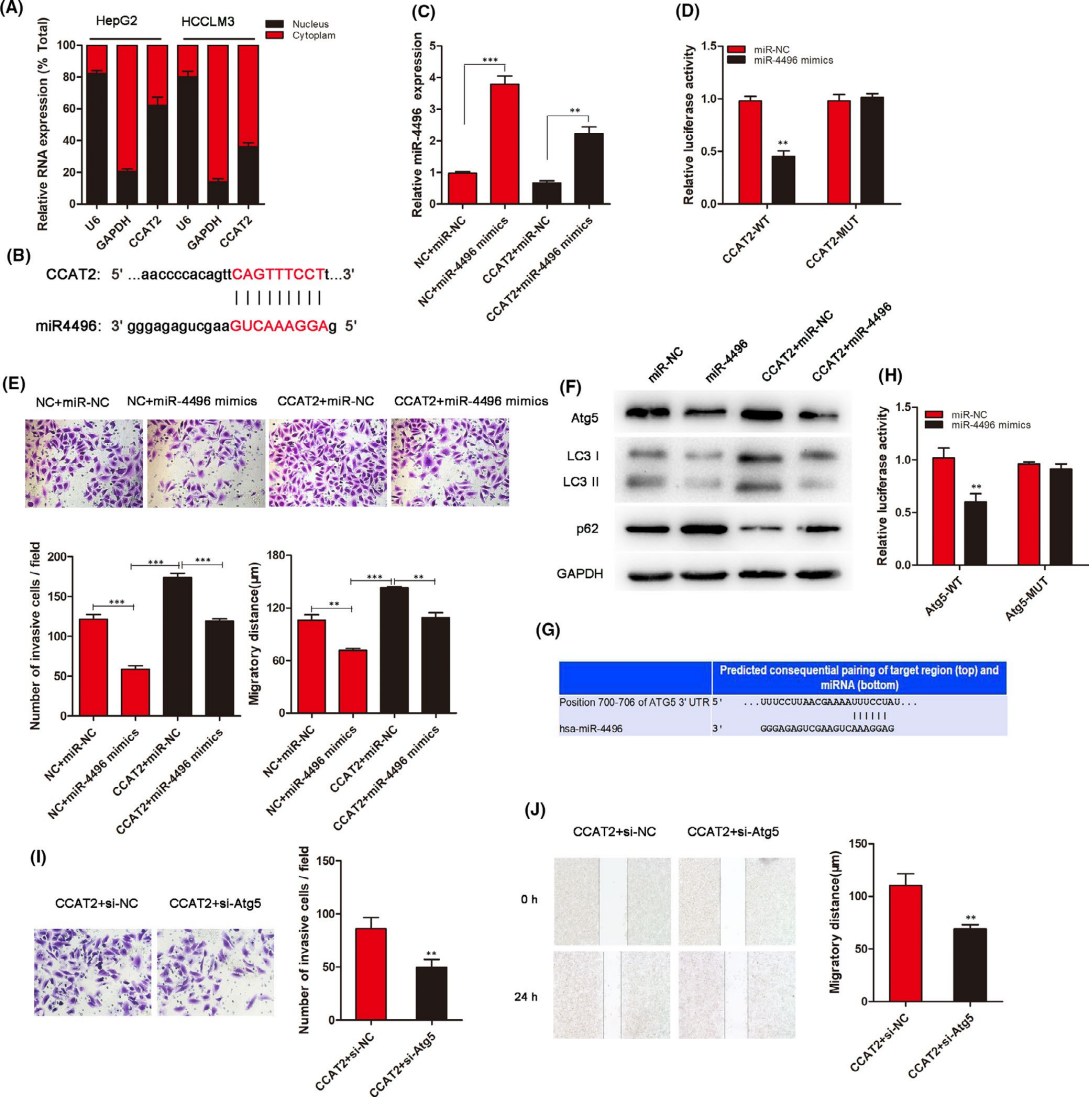
CCAT2 promotes migration and invasion by miR‐4496/Atg5 axis in HCC cell cytoplasm. (A) After nuclear and cytosolic separation, RNA expression levels were measured by qRT‐PCR. GAPDH and U6 were used as cytosolic and nuclear markers, respectively. (B) RegRNA2.0 database showed the binding sites between CCAT2 and miR‐4496. (C) MiR‐4496 expression was detected in HCCLM3 by qRT‐PCR after transfection with miR‐4496 mimics or combination with CCAT2. (D) Dual‐luciferase reporter assays demonstrated that miR‐4496 significantly reduced luciferase activity (wild‐type). (E) Overexpression of CCAT2 promoted migration and invasion of HCCLM3 cells, while cotransfection with miR‐4496 mimics could attenuate this effect influenced by CCAT2. (F) Western blot was used to detect the expression of Atg5, P62 and conversion of LC3‐I to LC3‐II influenced by miR‐4496 mimics and CCAT2. (G) Bioinformatic analysis predicted the potential binding of Atg5 to miR‐4496. (H) Luciferase reporter gene assays were performed to measure the luciferase activity in HCCLM3 cells. (I&J) Transwell and wound healing assays were used to explore the influences to invasion and migration of HCCLM3 cells when Atg5 was knocked down. ^**^ *p* < 0.01, ^***^ *p* < 0.001

To explore the mechanism for CCAT2‐mediated regulation, we performed a subcellular fractionation location assay to investigate CCAT2 location. As shown in Figure 4A, CCAT2 located both in the nucleus and in the cytoplasm. Bioinformatic prediction was performed (RegRNA2.0: http://regrna2.mbc.nctu.edu.tw/) to found miRNAs binding with CCAT2. miR‐4496 was the most sensitive RNA to bind to CCAT2 (Figure 4B; Figure S2). Through transfected with miRNA mimics, we furtherly confirmed the connection between them (Figure 4C). To confirm whether miR‐4496 was a direct target of CCAT2, a dual‐luciferase reporter system was conducted. As shown in Figure 4D, the luciferase activity was reduced significantly compared with the negative control by miR‐4496 mimic. We then explored the role of miR‐4496 in CCAT2‐promoted migration and invasion by cotransfecting HCCLM3 cells with CCAT2 and miR‐4496 mimics. The results showed that miR‐4496 reduced cell migration and invasion, while CCAT2 induced a higher migratory and invasive potential, which could be inhibited by miR‐4496 mimics (Figure 4E). What's more, a decreased conversion of LC3‐I to LC3‐II, reflecting inhibited autophagy activity, was observed when miR‐4496 mimics was cotransfected (Figure 4F). To validate the effects of miR‐4496 in autophagy, by the bioinformatic analysis (http://www.targetscan.org/), we predicted mRNA to interact with miR‐4496 and identified a putative binding site for Atg5 in the miR‐4496 sequence (Figure 4G). Luciferase assay revealed the combination between them furtherly (Figure 4H). As an autophagy regulator, Atg5 was involved in the malignant biological behaviour of HCC. As shown in Figure 4I,J, Atg5 knockdown could decrease the number of invasive cells promoted by CCAT2. These results suggested that CCAT2 affects HCC cell migration at least partly through miR‐4496/Atg5.

### 3.6 CCAT2 promoted progression of HCC by binding with ELAVL1

**FIGURE 5:**
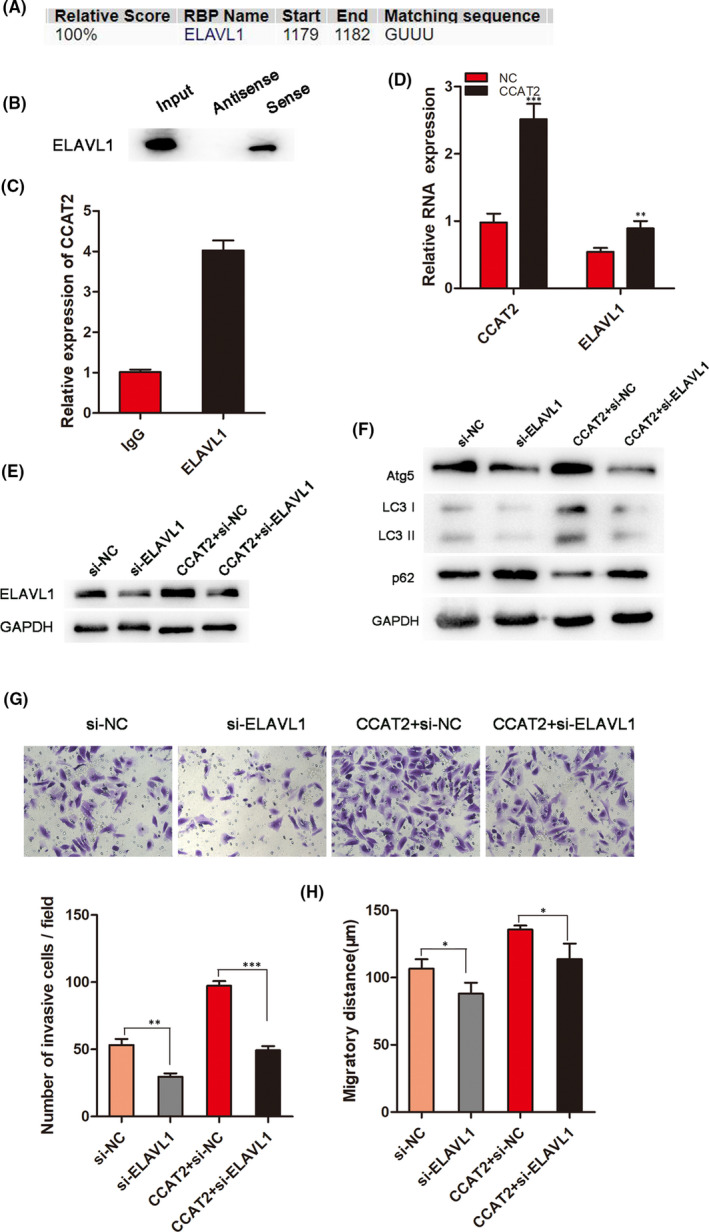
CCAT2 binds with ELAVL1 to promote progression of HCC. (A) RBPDB (http://rbpdb.ccbr.utoronto.ca/) database predicted the RNA‐binding proteins of CCAT2. (B) RNA pull‐down assay was conducted to analyse the ELAVL1 antibody. (C) RIP experiments showed that CCAT2 was enriched with ELAVL1‐immunoprecipitation relative to control IgG. (D) qRT‐PCR assay was used to indicate CCAT2 and miR‐4496 expression influenced by CCAT2. (E) Overexpressed CCAT2 promoted ELAVL1 expression detected by Western blot. (F) ELAVL1 knockdown decreased the expressions of Atg5 and LC3‐I conversion promoted by CCAT2. (G&H) Transwell and wound healing assays showed that CCAT2 promoted cell migration and invasion and ELAVL1 knockdown decreased the migrated and invasive cells induced by CCAT2. ^*^ *p* < 0.05, ^**^ *p* < 0.01, ^***^ *p* < 0.001

LncRNAs often exert their functions through RNA‐interacting proteins. Through bioinformatic analysis (http://rbpdb.ccbr.utoronto.ca/; http://pridb.gdcb.iastate.edu/RPISeq/references.php), we dentified a set of RBPs targeted by CCAT2, including ELAVL1 (Figure 5A), that has been reported to be involved in RNA regulation. To investigate the interaction between CCAT2 and ELAVL1, we conducted the RNA pull‐down assay to confirm that CCAT2 interacted with ELAVL1 (Figure 5B). Furtherly, RIP assay was performed to show the enrichment of CCAT2 in the ELAVL1‐immunoprecipitation and confirm the interaction of CCAT2 and ELAVL1 (Figure 5C). Moreover, overexpressed CCAT2 promoted ELAVL1 RNA expression detected by qRT‐PCR (Figure 5D), suggesting a regulatory role of CCAT2 in ELAVL1 expression. It is reported that ELAVL1 promoted autophagy by regulating expression of autophagy‐related proteins 5, 12 and 16.^23^ In our study, to investigate whether ELAVL1 affects autophagy, HCCLM3 cells were transfected with si‐ELAVL1, and the levels of autophagy‐related protein were assessed by Western blot (Figure 5E). The results showed that ELAVL1 knockdown decreased the expressions of Atg5 and LC3‐I conversion, while CCAT2 prompted this conversion (Figure 5F). This suggested that ELAVL1 might involve in autophagy. Next, we found that ELAVL1 silencing could decrease the migrated and invasive cells promoted by CCAT2 (Figure 5G,H). The above results illustrated that CCAT2 interacted with ELAVL1 to promote malignant biologic behaviour of HCC cells.

### 3.7 Expression of miR‐4496 and ELAVL1 in clinical specimens of HCC

**FIGURE 6:**
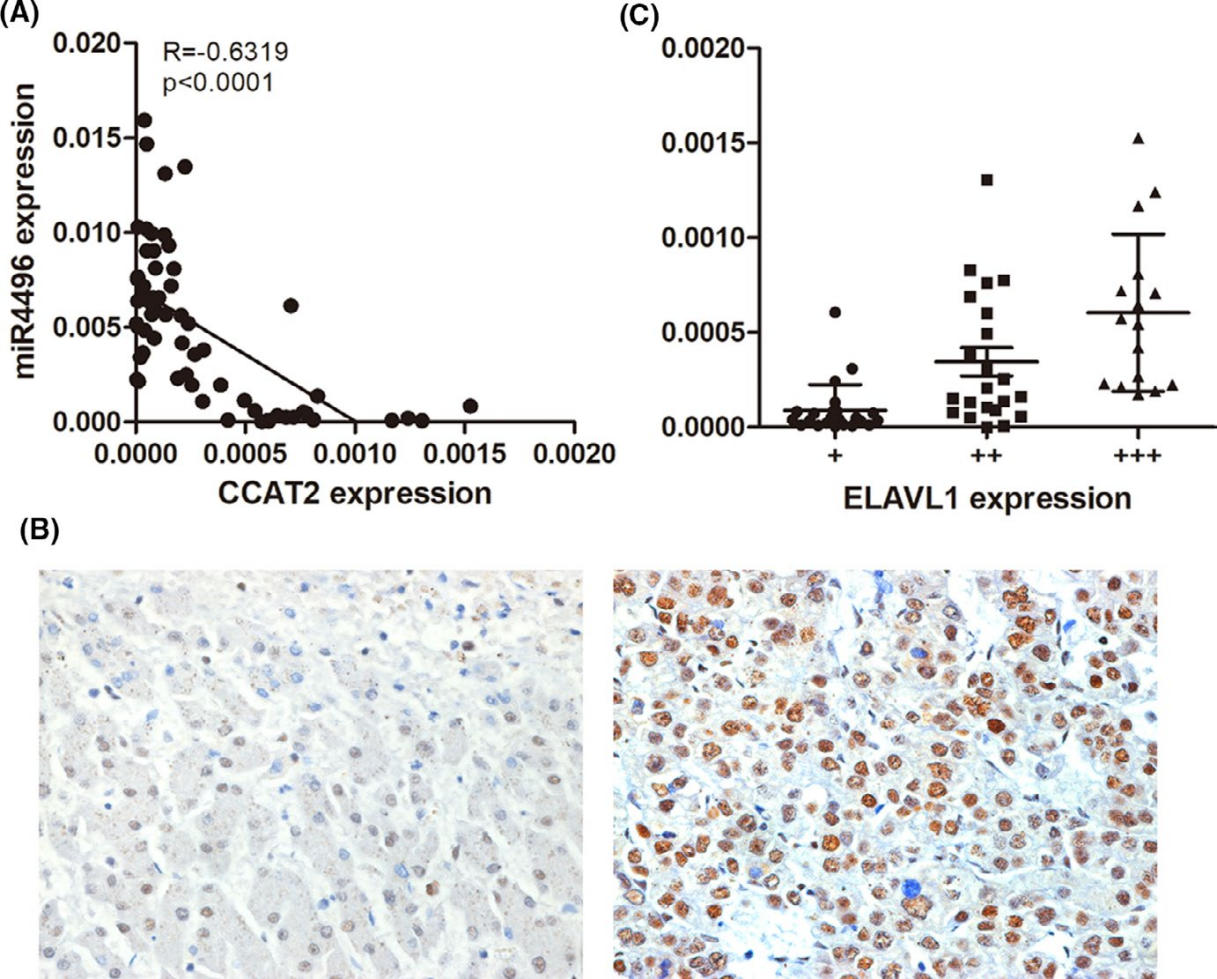
Correlation between miR‐4496 (ELAVL1) and CCAT2 in HCC patients' tumour tissue. (A) miR‐4496 and ELAVL1 expressions were determined by qRT‐PCR. There was an inverse correlation between them in HCC tissues (*R* = −0.6319, *p* < 0.0001). (B) ELAVL1 expression was mainly located in the nucleus of HCC tissue. (C) A scatter diagram showed an inverse correlation between the expression of CCAT2 and ELAVL1 in HCC tissue (original magnification ×200)

To further investigate the correlation between miR‐4496 (ELAVL1) and CCAT2 in HCC patients' tumour tissue, we examined miR‐4496 and ELAVL1 expressions by qRT‐PCR and IHC of 61 HCC specimens. As shown in Figure 6A, expression level of miR‐4496 was significantly upregulated and was negatively correlated with CCAT2 in HCC tissues (*R* = −0.6319, *p* < 0.0001). Furtherly, we compared ELAVL1 with CCAT2 expression level of HCC tissues. The results indicated that ELAVL1 expression was mainly located in the nucleus, and there was an inverse correlation between ELAVL1 and CCAT2 in these specimens (Figure 6B,C).

## 4 DISCUSSION

The regulatory role of autophagy in cancer metastasis is multifaceted with tumour‐suppressing or promoting functions, which is dependent on cell type and the tumour microenvironment.^24^ It is proposed that autophagy could encourage the progression of HCC through inhibiting tumour suppressors or contributing to chemoresistance.^25^, ^26^ The mechanisms involved in autophagy‐regulated metastasis were explored. Autophagy played a key role in focal adhesion dynamics during cancer metastasis by promoting a stem cell phenotype and production of autophagy‐dependent secreted factors. Rho GTPase‐mediated cytoskeleton remodelling also contributes to autophagy‐regulated metastasis.^27^ What's more, by regulation of epithelial mesenchymal transformation (EMT), autophagy modulates invasiveness of tumour cell.^28^ More than 30 Atg genes regulate autophagy and inhibition of particular Atgs could selectively inhibit autophagy at specific steps during autophagy occurrence. Our results established Atg5 as an important regulatory factor during cancer metastasis.

CCAT2 harbours the SNP rs6983267, which was reported to be associated with risk of many kinds of cancers. Studies have shown that CCAT2 might function as an oncogene.^29^, ^30^ Consistently, our study indicated that CCAT2 was highly expressed in HCC tissues and closely related with higher malignancy. A significant proportion of CCAT2 is mainly located in the nucleus and can be tissue‐ and cell‐type specific.^31^ We found that CCAT2 located both in the nucleus and in cytoplasm in HCC cells. LncRNA is emerging as important regulators of biological pathways and CCAT2 can influence cellular processes through different molecule mechanisms in the nucleus or cytoplasm. CCAT2 acts as sponge for some tumour suppressor miRNAs, thus promoting cancer evolution. It is reported that CCAT2 could stimulate the expression of miR‐17‐5p and miR‐20a by interacting with TCF7L2.^11^ CCAT2 inhibited miR‐145 maturation and increased metastatic potential of cancer cells.^31^ Moreover, upregulation of CCAT2 promoted tumorigenesis process *via* alterations in several cancer‐related pathways including Wnt and MAPK pathways.^32^, ^33^ In our study, we found that CCAT2 could regulate autophagy‐related metastasis by interacting with miR‐4496.

In addition to the inhibition of miRNA, lncRNAs perform a plethora of cellular functions by interacting with one or more RNA‐binding proteins (RBPs).^34^ The RNA‐binding protein ELAVL1 is predominantly nuclear but translates to the cytoplasm *via* a nucleocytoplasmic shuttling domain when the cell receives one of various stimulations.^35^ By phosphorylation or methylation, ELAVL1 binds to a subset of mRNAs and influences their stability or translation.^36^ Involvement of ELAVL1 in autophagy was also investigated. Lan Xiao found that knockdown of ELAVL1 prevented rapamycin‐induced autophagy.^37^ Soumasree De indicated that ELAVL1 was one of the key regulatory mechanisms of starvation‐induced autophagy in breast cancer cell MCF‐7.^38^ Another two studies showed that ELAVL1 regulated autophagy by modulating Atgs translation, and augmented expression of ELAVL1 and Atgs might participate in the malfunction of autophagy.^23^, ^39^ Our results found that ELAVL1 involved in the regulation of CCAT2‐promoted autophagy in HCC cells through Atg5. In a word, our study suggests that CCAT2 promotes HCC invasion by autophagy induction. miR‐4496/ELAVL1‐Atg5 signalling pathways facilitate the process. Findings in this study may contribute to reveal molecular mechanism associated with progression of HCC, thus providing new potential therapeutic targets.

## 5 CONCLUSIONS

CCAT2 enhances autophagy‐related invasion and metastasis *via* regulating miR‐4496 and ELAVL1 in HCC. Our data demonstrated the important roles of CCAT2 in HCC progression and might serve as a target for HCC therapy.

## CONFLICT OF INTEREST

The authors declare that they have no competing interests.

## AUTHOR CONTRIBUTIONS

**Jing Shi:** Data curation (lead); Formal analysis (lead); Methodology (supporting); Project administration (lead); Resources (supporting); Supervision (supporting); Validation (supporting); Visualization (supporting); Writing‐original draft (lead). **Cao Guo:** Data curation (supporting); Formal analysis (supporting); Investigation (equal); Methodology (lead); Project administration (supporting); Software (lead). **Junli Ma:** Conceptualization (lead); Funding acquisition (lead); Investigation (supporting); Project administration (supporting); Resources (lead); Software (supporting); Supervision (lead); Validation (lead); Visualization (lead); Writing‐review & editing (supporting).

## Supporting information

Fig S1

Fig S2

Tab S1

Tab S2

## Data Availability

The datasets used and/or analysed during the current study are available from the corresponding author on reasonable request.
