# Supplementary figures and images for: CCAT2 enhances autophagy‐related invasion and metastasis via regulating miR‐4496 and ELAVL1 in hepatocellular carcinoma
Source: J Cell Mol Med. 2021 Aug 19;25(18):8985–96. doi: 10.1111/jcmm.16859 (PMC8435435; doi:10.1111/jcmm.16859)

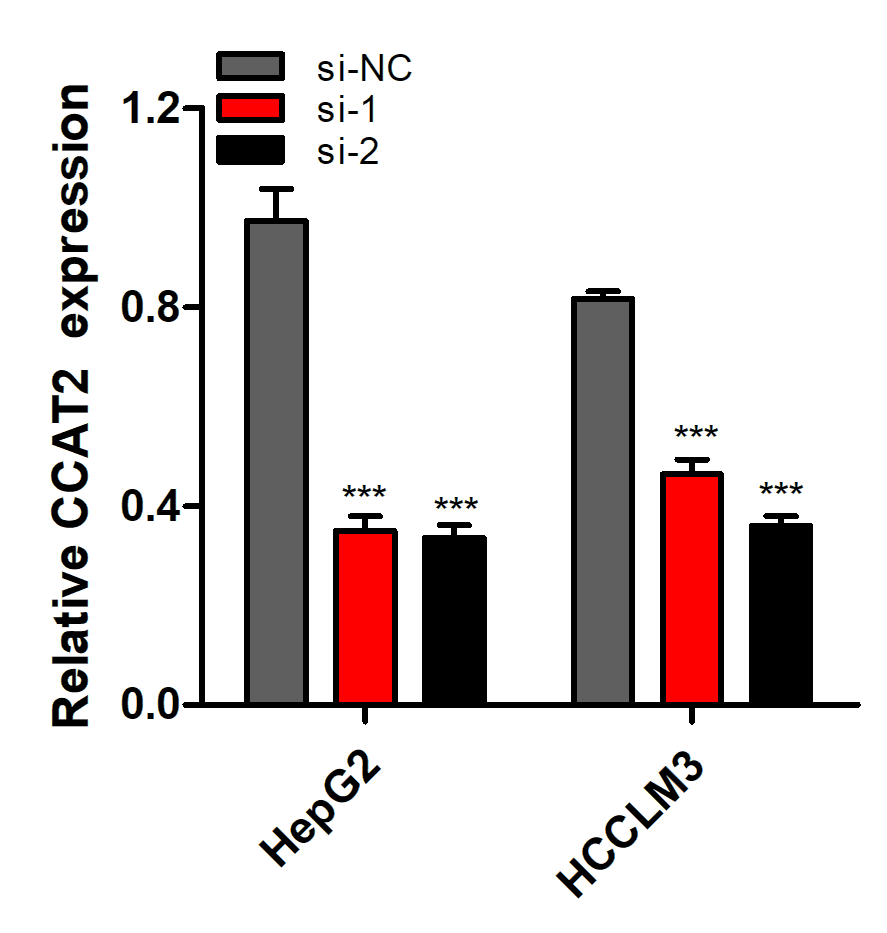

Supplement: Supplementary file 1 — Fig S1 [file JCMM-25-8985-s004.tif]

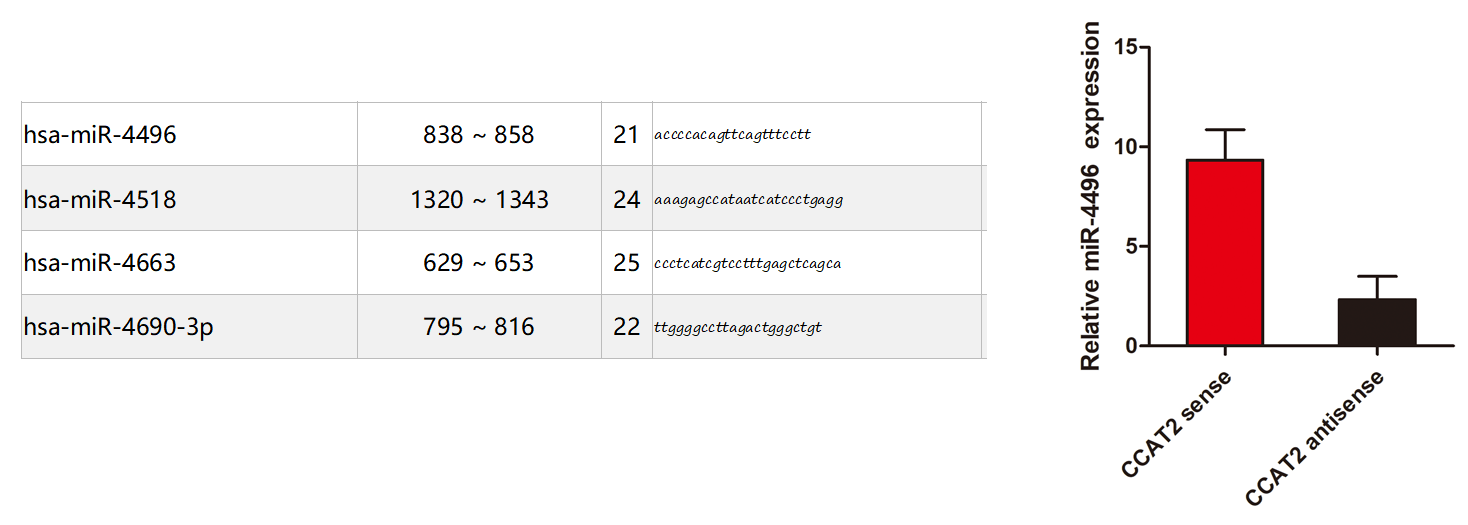

Supplement: Supplementary file 2 — Fig S2 [file JCMM-25-8985-s001.tif]
