# Supplementary material for: CCAT2 enhances autophagy‐related invasion and metastasis via regulating miR‐4496 and ELAVL1 in hepatocellular carcinoma
Source: J Cell Mol Med. 2021 Aug 19;25(18):8985–96. doi: 10.1111/jcmm.16859 (PMC8435435; doi:10.1111/jcmm.16859)
Supplement: Supplementary file 3 — Tab S1 [file JCMM-25-8985-s002.docx]

**Table S1** The siRNA sequences of CCAT2 and ELAVL1.

| **Names** | **Sequences** |
| --- | --- |
| si-NC | TTCTCCGAACGTGTCACGT |
| si-CCAT2-1 | GUAACCUCUUCCUAUCUCATT |
| si-CCAT2-2 | UUAAAUUGCAGAGUUGCACUU |
| si-ELAVL1 | AAGAGGCAATTACCAGTTTCA |
