# Supplementary material for: CCAT2 enhances autophagy‐related invasion and metastasis via regulating miR‐4496 and ELAVL1 in hepatocellular carcinoma
Source: J Cell Mol Med. 2021 Aug 19;25(18):8985–96. doi: 10.1111/jcmm.16859 (PMC8435435; doi:10.1111/jcmm.16859)
Supplement: Supplementary file 4 — Tab S2 [file JCMM-25-8985-s003.docx]

**Table S2：****Oligonucleotide sequences of quantitative real-time PCR primers.**

| **Gene** | **Forward sequence** | **Reverse sequence** | |
| --- | --- | --- | --- |
| CCAT2 | 5’-GGTGCTCCAGGCAATAACT-3’ | | 5’- CTTCCTACAGGCCCAAACAT -3’ |
| miR-4496 | 5’-AAAGGTACCCCACCCCGGCTAATTTTG  TATTTATAG -3’ | 5’-TTTAAGCTTCTGATGTATGCAAAGCA  CCTACCAC-3’ | |
| ELAVL1 | 5’-TGTTCTCTCGGTTTGGGCGGAT -3’ | 5’-TCTTCTGCCTCCGACCGTTTGT -3’ | |
| GAPDH | 5’-tgtctggcacattggacatt -3’ | 5’-GCACCGTCAAGGCTGAGAAC -3’ | |
